# Food Insecurity and Food Label Comprehension among Libyan Migrants in Australia

**DOI:** 10.3390/nu13072433

**Published:** 2021-07-15

**Authors:** Reima Mansour, James Rufus John, Pranee Liamputtong, Amit Arora

**Affiliations:** 1 School of Health Sciences, Western Sydney University, Campbelltown Campus, Locked Bag 1797, Penrith, NSW 2751, Australia; a.arora@westernsydney.edu.au; 2 Health Equity Laboratory, Campbelltown, NSW 2560, Australia; james.john@westernsydney.edu.au; 3 Department of Nutrition, Faculty of Public Health, Benghazi University, Benghazi, Libya; 4 School of Psychiatry, University of New South Wales, Sydney, NSW 2052, Australia; 5 South Western Sydney Local Health District, Liverpool, NSW 2170, Australia; 6 Ingham Institute of Applied Medical Research, Liverpool, NSW 2170, Australia; 7 College of Health Sciences, VinUniversity, Gia Lam District, Hanoi 100000, Vietnam; pranee@latrobe.edu.au or; 8 Translational Health Research Institute, Western Sydney University, Locked Bag 1797, Penrith, NSW 2751, Australia; 9 Discipline of Child and Adolescent Health, Sydney Medical School, Faculty of Medicine and Health, The University of Sydney, Westmead, NSW 2145, Australia; 10 Oral Health Services, Sydney Local Health District and Sydney Dental Hospital, NSW Health, Surry Hills, NSW 2010, Australia

**Keywords:** food availability, food affordability, food accessibility, minorities, nutrition knowledge

## Abstract

Food security among migrants and refugees remains an international public health issue. However, research among ethnic minorities in Australia is relatively low. This study explored the factors that influence the understanding of food labelling and food insecurity among Libyan migrants in Australia. An online survey was completed by 271 Libyan migrant families. Data collection included the 18-item US Household Food Security Survey Module (for food security) and a question from the Food Standards Australia New Zealand Consumer Label Survey (for food labelling comprehension). Multivariable logistic regression modelling was utilised to identify the predictors of food label comprehension and food security. Food insecurity prevalence was 72.7% (*n* = 196) while 35.8% of families (*n* = 97) reported limited food label understanding. Household size, food store location, and food affordability were found to be significantly related to food insecurity. However, gender, private health insurance, household annual income, education, and food store type and location were found to be significantly related to food labelling comprehension. Despite the population’s high educational status and food labelling comprehension level, food insecurity remained an issue among the Libyan migrants. Policy makers should consider the incorporation of food label comprehension within a broader food security approach for migrants.

## 1. Introduction

Food insecurity is more than the availability of sufficient quantities of food. It exists “whenever the availability of nutritionally adequate and safe foods, or the ability to acquire acceptable food in socially acceptable ways, is limited or uncertain” [1]. Food insecurity is often seen in residents of low- and middle-income countries. However, food insecurity is also prevalent in high-income countries, with higher rates seen among some population groups. In the USA, 10.5% of households were reported to be food insecure. This meant that they did not have access to food for an active and a healthy life for all household members [2]. The most recent Canadian national estimate suggests that one in eight households do not have adequate access to food [3]. In Australia, the prevalence of food insecurity was approximately 4% during the period 2011–2012 [4]. However, a recent systematic review reported that food insecurity in Australia ranged between 2% and 90% [5] among different population groups. The majority of studies included in the systematic review used a single-item measure to ascertain food insecurity, with results ranging from 2% among older Australians to 76% among remote Indigenous communities [5]. A few studies used the seven-item USDA measure and reported food insecurity to be as high as 48% among university students and up to 90% among asylum seekers in Australia [5]. 

Australia is among the world’s top seven countries for food affordability [6]. It is also one of the most food-secure countries, ranking twelfth on the Global Food Security Index [6]. Australia produces large quantities of high-quality food to meet the needs of its population and sustain vigorous exports [7,8]. Food for domestic consumption is further supplemented by imports. Additionally, higher employment levels and an income support safety net ensure that food is affordable and accessible to most Australians. According to the Household Expenditure Survey 2015–2016, Australians spend approximately 16.1% of their average income on food and non-alcoholic beverages [9]. Despite this, research has shown that some families still find it difficult to access and afford nutritious food [10,11,12,13]. 

Many factors have been identified with food insecurity among both general and migrant populations. According to an Australian systematic review, factors associated with food insecurity among refugees include unemployment, low income, limited time available for shopping, and the low availability and high cost of culturally appropriate foods [14]. Food insecurity, specifically among migrant or refugee families, is exacerbated by isolation [13]. However, there is limited research on food labelling and its impact on food insecurity among the general Australian population. This is further scarce among migrant communities in Australia [15,16]. A recent Australian study [15] reported that food insecure participants were less likely to comprehend food labels compared to their counterparts. Furthermore, food secure participants were twice as likely to report that they had a healthy diet than those who were food insecure. In an Australian study, food insecure participants were 1.4 times less likely to use nutrition information panel [16]. However, factors such as recent migration and language other than English that might contribute to this finding were not explored. Other limitations include the under-representation of vulnerable populations such as those living in remote areas, those with low levels of literacy, and culturally and linguistically diverse (CALD) groups [16]. There exist significant gaps in the literature related to food security and label comprehension in migrant populations. 

Food labelling should be given critical attention as it has a significant impact on food security, overall health, and wellbeing [17]. Nutritional labelling knowledge predisposes consumers to use food labels and significantly affects their purchasing behaviour [15]. Several studies indicate that nutrition information may help consumers select healthier products more easily [18,19,20,21]. Both nutrition panels and food fact labels have been shown to be associated with healthier food choices [21]. However, consumers whose choices were driven by price were found to be less likely to read labels [21]. It is rather concerning that a gap exists in the literature between intention and actual behaviour [21]. In addition, no studies have specifically addressed the experience of migrant populations to date. 

The usefulness of the nutrition labels depends on language comprehension and an understanding of the specific vocabulary and associated concepts with which even native English speakers struggle. A US study among CALD migrants reported difficulty understanding servings per package and percentage of daily intake value [22]. Another US study among Hispanic migrants noted a change in eating habits since migration with the consumption of fresh fruit and vegetables falling substantially due to perceived higher cost and lower quality [23]. Moreover, the participants recommended education in reading and interpreting food labels to improve their food habits and nutrition [23]. 

Australia has been considering the adoption of a strategy to improve and simplify food labelling in an effort to improve purchasing behaviours and address poor dietary intakes [24]. It is known as the “traffic light system” for the front of the package with three colours used to signify the foods’ relative acceptability. The colour red, for example, is to be used to warn consumers of low nutritional value (i.e., as an indicator that the food is high in kilojoules (KJ), saturated fat, added sugar and/or salt, and low in fibre). It also indicates that such foods should be consumed rarely and in small amounts [17,18,24]. Already adopted in the UK, the system has received support elsewhere in research [17]. However, it has not yet been adopted nationally across all states in Australia [24].

While interest in food labels and food security may be high among migrant groups, recent scoping reviews [19,25] suggest the actual understanding of the topic is low. This was especially the case among Middle Eastern–North African (MENA) populations. Australia and New Zealand have limited nutrition research among Arabic-speaking immigrants and refugees [19]. However, North America is substantially ahead of Europe in this type of research. To our knowledge, no research has been conducted into food-insecure households’ self-reported understanding of food labels among Libyan migrants.

The majority of the Libyan population currently residing in Australia had voluntarily entered the country as students. They were forced to seek refuge in Australia due to a revolution that begun in Libya in 2011 [26]. The Libyan population also comprises families who have lived in Australia for more than 20 years [27]. The situation of food security in Libya has become impaired due to the prolonged conflict [28]. Moreover, food insecurity levels during the period 2014–2019 were reported to be five times higher than the rates during the period 2003–2009 [28,29]. There are only a few studies on the nutritional status of MENA migrants and refugees in high-income countries [19,25]. MENA migrants maintain their cultural identities through the consumption of special religious and traditional foods [30]. These food preferences and ethnic practices might therefore impair their overall well-being [19,30]. As most Libyans migrants are Muslim, they may wish to observe particular dietary guidelines (which identify certain foods as legal or *halal* and others not) as part of their religious practice [30,31]. It is posited that despite high levels of education [31], comprehending the specific language of food labelling could prove a challenge to them and affect food security.

This study seeks to address the knowledge gap regarding food security among Libyan migrant families in Australia. Given the ongoing program of migration to Australia, such studies are particularly relevant. This study explores the relationship between food insecurity, understanding of food labels, and other factors such as food choices, food access, purchasing behaviours, and economic and sociodemographic factors. It is the first study of its type among a specific migrant population group that is often under-represented in nutrition research. A few prior Australian studies have measured label comprehension in the general population but not among minority populations [15,16]. No studies have explored a possible relationship between label comprehension and food insecurity in a minority migrant population, both in Australia and internationally.

The aim of this study is to identify the prevalence of food insecurity among Libyan migrant families in Australia and the level of food label comprehension. It also seeks to determine whether and to what extent there is an association between food label comprehension and food security and sociodemographic factors.

## 2. Methods

### 2.1. Participants and Recruitment 

A cross-sectional design using an online survey was conducted to determine the level and prevalence of food insecurity as well as comprehension of food labels among Libyan families in Australia. Participants were drawn from a population of 500 Libyan migrant families (comprising more than 2810 people, including children) currently estimated to be living in Australia [31,32]. The majority resided in New South Wales (NSW) and Victoria [31].

The Australian Libyan Association Inc. and the Libyan Embassy in Australia assisted in contacting migrants by email, and offering them the opportunity to participate via a link attached with the invitation. The study also used an online version that was linked to the social media presences of Libyan immigrant groups (Facebook, Instagram, and WhatsApp) that participants were also able to access. Data collection was undertaken between October 2019 and February 2020. A statement was included which indicated that participants implied their consent by accessing and completing the online survey.

### 2.2. Sampling

“Snowball” sampling, a form of convenience sampling, was selected for recruiting participants for this cross-sectional study. This non-probability sampling strategy was used because of the ease of access to the target population and its time- and cost-effective nature [33]. “Snowball” sampling also increases the potential to maximise sample size [34,35]. A sample size calculation was undertaken based on an estimated total possible population of 500 families (with a margin of error of 4%, assuming 50% positive response and a confidence level of 95%) [36,37]. This was to estimate the minimum number of respondents required for the results to have sufficient statistical power. The minimum sample size required to ensure a margin of error of ±5% was calculated to be 235 families. 

### 2.3. Data Collection 

Participants were asked for information regarding their food access, food choices, understanding of food labelling, and food purchasing behaviours. They were also asked about their experience of food insecurity since they started living in Australia. Questions regarding socio-demographic and socio-economic factors were also included. The survey took approximately 30 min to complete. 

The questionnaire used in this study includes the United States Department of Agriculture (USDA) Household Food Security Survey Module (USDA HFSSM). It is an 18-item scale derived from the USDA Community Food Security Assessment Toolkit and is used to measure food insecurity for households with children [38]. As a comprehensive and validated tool for measuring food insecurity, it has previously been used in large-scale research in countries including the USA, Canada, and Australia [2,3,12,39].

To examine the understanding of food labels, an additional question was adopted from the Food Standards Australia New Zealand (FSANZ) Consumer Label Survey [40]. The question—“How well do you understand information in ingredient lists?” elicited the self-reported level of respondents’ understanding of food product labels. Labels include a list of ingredients in descending order of percentage of the contents and some additives that are identified by a number and nutrition information panel. The latter lists energy (KJ) protein, fat, carbohydrates, sugars, dietary fibre, and sodium, and presents each as a percentage of recommended daily intake (RDI) based on average adult intake [17,38]. 

Additional questions were developed by the researchers in this study regarding special food requirements (including cultural and religious food needs) and food purchasing behaviours. An online version of the survey was designed and data were collected using Qualtrics (online survey software, Provo, UT, USA) [41]. 

### 2.4. Outcomes

For food security, the 18 questions of the USDA HFSSM were combined into a single overall measure called the “food security scale”. These were binarily coded as either “affirmative” (indicating food insecurity) or “negative” (indicating food security). For food labelling, the FSANZ question inquired how well the respondent understood the information in ingredient lists (food labels) [40]. Response options were “not well at all,” “slightly well,” “moderately well,” “very well”, and “extremely well.” For this question, “moderately well,” “very well “, and “extremely well” were combined into one category coded as “affirmative” to obtain an estimate of those who well-understood food labels. Responses “not well at all” or “slightly well” were combined into a single category indicating a limited understanding of food labels and coded as “negative”.

Socio-demographic data collected comprises the respondent’s age (in years), gender (man or woman), length of stay (in years), their English language proficiency (low, intermediate, high), education (vocational/high school/less, undergraduate university, postgraduate university), number of family members, and the postcode classification of the place of residence (urban, rural). For the socio-economic attributes, data collected comprised employment status (yes, no), annual income (<AUD 40,000, ≥AUD 40,000), and occupation status (managerial, professionals or skilled/unskilled, pensioner/unemployed). The residential location was further stratified on a socioeconomic/disadvantage basis. The suburbs were divided into five categories (1–2, 3–4, 5–6, 7–8, 9–10) using the residential postcode as per the Index of Relative Socioeconomic Advantage and Disadvantage (IRSAD). An Australian Bureau of Statistics (ABS) product, the IRSAD, ranks areas in Australia on a continuum from most disadvantaged to most advantaged [42]. 

Information about participant’s perceived barriers to food security was collected. These barriers included the food store type they accessed (supermarket, or local food store and supermarket, or local store) and the location of food stores. Information was gathered on other perceived barriers. These included the price of food, the availability of healthy and culturally appropriate foods (always/occasionally, seldom), and food quality which might be compromised by a desire to consume culturally appropriate foods that are available but not of the best standard. 

### 2.5. Statistical Analysis 

The data were assessed for quality and the responses were checked for missing data. Incomplete surveys were excluded from the analysis. Descriptive data analysis included the estimation of the overall prevalence of food insecurity, stratified by gender and age. Continuous variables are shown as mean and standard deviation (SD). However, the frequency counts of categorical variables are shown as percentages. Univariable logistic regression analysis was then conducted to ascertain the factors independently associated with food insecurity and understanding of food labels. Multivariable logistic regression analysis was conducted to identify the relationship between the socio-demographic and socio-economic variables and the two main outcome variables (food security using the 18-item measure and the understanding of food labelling using the FSANZ question). Binary logistic regression using backward stepwise procedure analysis was used to develop models, remove non-significant variables, and predict both food insecurity status and the understanding of food labelling with an adjusted odds ratios (AOR) and 95% confidence intervals. Finally, variables that had a significant statistical association (*p* < 0.05) with food insecurity and understanding of food labelling remained in the final model.

Multicollinearity in logistic regression was used to investigate any significant collinearity among variables prior to entering them into the models. Version 26 of IBM’s Statistical Package for the Social Sciences (SPSS) software was used to analyse data gathered from the questionnaire (Armonk, NY, USA: IBM Corp.).

## 3. Results

### 3.1. Baseline Characteristics

**Figure 1:**
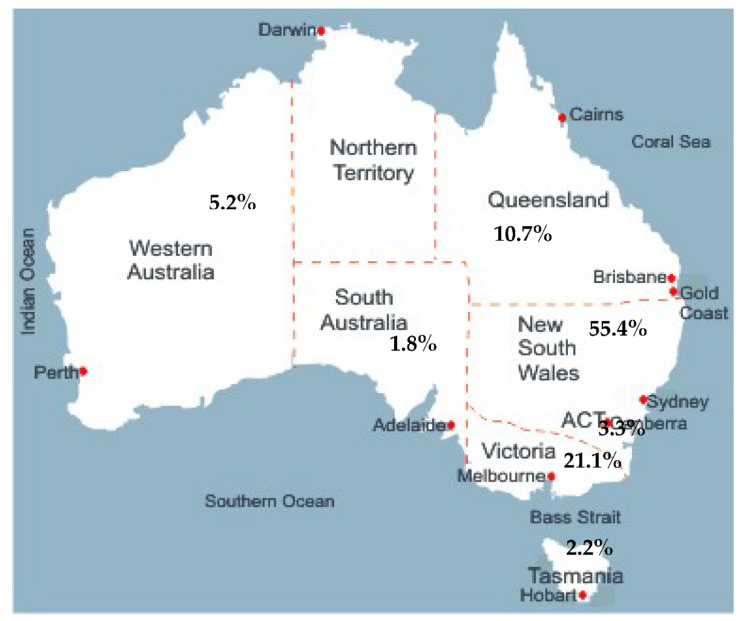
Geographic distribution of the participants’ states of residence in Australia.

The demographic characteristics of the study population and food experiences related to specific cultural and/or religious food requirements are presented in Table 1. Of the 500 families that comprise the Libyan population in Australia, 303 families from all states and territories of Australia began the online questionnaire. Of the 303 families, 32 failed to complete it. A total of 271 participants (54.2%) fully completed the survey and their data were included in the final analysis. The mean age of the respondents was 38 ± 7 years with a slight majority of women (60.5%) among the survey respondents. Most of the respondents (83.4%) were educated at the university level (undergraduate or postgraduate), with 57.2% of respondents having studied at the postgraduate university level. Families ranged in size from two to ten members, with a mean of five members per family. The majority of participants reported residing in NSW (55%) followed by Victoria (21%) (Figure 1). Pensioners or unemployed individuals formed about 65% of respondents. Approximately 33% of respondents resided in postcodes recognised as highly or most disadvantaged areas (21% and 11.6%, respectively). 

In terms of food requirements, 98.5% of respondents preferred *halal* food. Only 5.2% of participants had vegetarian/vegan preferences or required specific food due to allergies. Some respondents (28%) purchased supplies solely from supermarkets while others (27.3%) bought solely from local food stores (such as a baker, butcher, or store selling traditional foods). About 44% of respondents purchased their food from both supermarkets and local food stores to satisfy their diverse requirements. In terms of ease of access, 70% of respondents found it difficult to obtain culturally appropriate foods. Another barrier to obtaining food of sufficient quality and quantity was product price, with 60.5% of respondents finding this to be the case. Moreover, 42% of respondents reported facing some difficulty getting to and from the shops with issues around the location of food stores and transport availability between their residence and food stores. 

Understanding of food labels was determined by using a question adopted from the FSANZ Consumer Label Survey. In this study, 35.8% (*n* = 97) of the participants had a limited understanding of food labels. Determined using the 18-item measure, the prevalence of food insecurity was found to be 72.7% (*n* = 196), with about three in four families reporting being food insecure (Table 1).

Univariable analysis for the understanding of food labels (Table 2) demonstrated that elderly persons had higher odds of having a limited understanding of food labels (OR = 1.06; 95% CI 1.02, 1.10). Increased length of stay was associated with 11% lower odds of limited understanding of food labels (OR = 0.89; 95% CI 0.81, 1.00). Compared to participants with a “low” level of English language proficiency, those reporting to have “intermediate” and “high” level of proficiency had about 50% lower odds of having a limited understanding of food labels (OR = 0.45; 95% CI 0.22, 0.92 and OR = 0.48; 95% CI 0.28, 0.91, respectively). 

Participants with a postgraduate university education (about 79%) had a greater understanding of food labels than those with a lower level of education (OR = 0.21; 95% CI 0.10, 0.43). In terms of the socio-economic attributes, respondents with higher income (≥AUD 40,000) were more likely to understand food labelling (OR = 0.27; 95% CI, 0.14, 0.53). Additionally, unemployed participants were more likely to have a limited understanding of food labels (OR = 1.80; 95% CI 1.06, 3.06) and as were people with no private health insurance (OR = 0.52; 95% CI 0.30, 1.00).

### 3.2. Univariable Analysis of Food Security

In relation to the socio-demographic factors (Table 3), an increased risk of food insecurity (4%) was associated with an increase in age (OR = 1.04; 95% CI 1.0, 1.08). Moreover, large families had a 27% increased risk of being food insecure (OR = 1.27; 95% CI 1.07, 1.49) compared to small families. Higher annual income was associated with a lower risk of food insecurity (OR = 0.53; 95% CI 0.28, 1.01) than lower annual income. Families living in highly disadvantaged areas (deciles 5 and 6) were three times more likely to be food insecure (OR = 3.00; 95% CI 1.01, 8.85) than those living in highly advantaged areas.

Several issues related to food purchasing behaviours were found to be highly associated with people being food insecure. The location of food stores, availability of healthy food, availability of culturally appropriate foods, and price of food, were found to be barriers to obtaining culturally appropriate foods. The quality of food and food storage, as barriers to obtaining preferred foods, were also associated with being food insecure. A limited understanding of food labels was far more likely to be associated with food insecurity than a good knowledge of food labelling.

### 3.3. Multivariable Logistic Regression for Demographic and Socio-Economic Variables, and Food Purchasing Behaviour in Relation to Food Labelling

Women demonstrated a greater understanding of food labelling than men (AOR = 0.50; 95% CI 0.25, 0.99). Respondents with postgraduate university education were far less likely to have a limited understanding of food labelling than those with lower education (AOR = 0.27; 95% CI 0.11, 0.70). Those with higher income (≥AUD 40,000) were more likely to understand food labelling (AOR = 0.34; 95% CI 0.17, 0.72). Likewise, those with no private health insurance had lower odds of a limited understanding of food labelling (AOR = 0.41; 95% CI 0.19, 0.91). People who bought food only from local stores had higher odds of having a limited understanding of food labels (AOR = 1.89; 95% CI 0.81, 4.40). Participants who got their food requirements from both supermarkets and local food stores appeared to have less likelihood of having a limited understanding with about 50% lower odds (AOR = 0.51; 95% CI 0.23, 1.13). Moreover, people who found the location of food stores to be a barrier to getting their food were more likely to have a limited understanding of food labels (AOR = 2.05; 95% CI 1.03, 4.1).

### 3.4. Multivariable Logistic Regression for Demographic and Socio-Economic Variables, and Food Purchasing Behaviour in Relation to Food Security

Large families were associated with a 35% higher risk of food insecurity (AOR = 1.35; 95% CI 1.11, 1.63). Families that cited the location of food stores as a barrier to accessing food had four times higher odds of food insecurity than those that had no problem with the location of food stores (AOR = 4.53; 95% CI 2.44, 8.40). Families that indicated the price of food as a barrier were approximately four times more likely to be food insecure (AOR = 3.71; 95% CI 2.02, 6.80) when compared with families that did not consider the price as a barrier.

## 4. Discussion

This study used the 18-item USDA measure to determine food insecurity among Libyan migrants in Australia. The prevalence of food insecurity was established on the basis of a household having experienced food insecurity (with or without hunger). The prevalence of food insecurity estimated in this study was 72.3%. This was significantly higher than that found in a recent study that reported a prevalence rate of 26% among CALD migrants in Tasmania, Australia [10]. The level of food insecurity found in this study was consistent with those reported in other studies among migrant and refugee populations in Australia [11,13,14] and in other high-income countries [39,42,43,44,45,46,47,48]. A recent systematic review on food insecurity among Australian refugees reported that food insecurity ranged from 35% to 90%, with 11% to 40% of the population having experienced severe hunger [14]. Research in other developed countries (including Canada, the USA, Norway, Germany, and other European countries) also found that the existence of hunger among such populations ranged from 37% to 93% [39,44,45,46,47,48]. 

Food insecurity may be linked to a number of socio-demographic and other characteristics of the population surveyed. These include family size, store location, and food prices. In this study, on average, larger families were more food insecure than smaller families or households. A similar pattern of results was obtained in other research among similar populations [49,50]. In contrast to our findings, some studies demonstrated that food insecurity status is negatively related to family size [2,51]. These studies suggest that families receiving government assistance may offset disadvantages otherwise associated with increased family size (such as lower per head income). In terms of food security barriers, our study found that the location of food stores and the price of food were highly significant. These results were consistent with prior studies [16,52,53,54,55] that have shown that food secure people are likely to compare prices to buy healthy food at the best prices. In contrast to other findings, a Tasmanian study found that less than 10% of persons in a similar population reported going without food. When this occurred, factors included a lack of affordability (high food price/insufficient funds), distance to shops, limited availability and high cost of culturally appropriate foods in Tasmania [52]. However, food labelling and its impacts were not considered [52]. 

In terms of the respondents’ understanding of food labels, our study found that more than one-third of the participants had a limited understanding of food labels. This was far higher than the level determined in a recent study of the general Australian population. In the Australian study, only about one in ten participants were found to have a limited understanding of food labels [40]. This finding shows the marked imbalance among the general Australian population and ethnic minority groups. Therefore, it is important for policymakers to examine this imbalance and provide effective solutions to address it.

Key factors identified as associated with the understanding of food labels were gender, annual income, and education. This could be attributed to the positive association between education and income [56]. Private health insurance appeared to be linked to comprehension, but it is likely due to its association with higher-income families. Similar observations were reported among migrants in the US, where food label skills were associated with higher education, higher income, and gender (men) [22]. Our findings partly align with a study conducted among the general population in six European countries (United Kingdom, France, Sweden, Germany, Poland, and Hungary) [57]. Education and gender were found to be related to nutrition comprehension in this European study, while occupational status was not. It should be noted that the term “social grades” was used as the measure of socio-economic status and annual incomes were not recorded. However, social grade or socio-economic status is closely related to the type of employment, with higher incomes associated with higher social grade and employment. In our study, occupational status was not related to the comprehension of labels, although income was. 

In terms of the use of labels in the European study [57], women as well as persons of higher social grades had a greater tendency to use nutrition panel information than men or those of lower social grades. Our study also found that women, as well as those on higher incomes or with higher education, were more likely to understand food labelling, but no such relationship with occupational status was found. More highly educated people are more likely to secure higher incomes which enable them to afford quality foods, including fresh food and imported culturally important food. Moreover, they are more likely to be able to understand food labelling that will enable them to access to accurate food knowledge. In the European study [57], although comprehension was higher in Sweden, Germany, and the UK than in the remaining countries, comprehension was higher than use across all six countries. This may be attributable to the role played by motivation which is needed to transform comprehension into use. Other factors may include the respondents’ interest in healthy eating, their knowledge of nutrition, and their social grade [57]. The question of the degree of transference of such self-reported knowledge into action could be the subject of a more detailed study in migrant populations in Australia.

In North America, a study among recent Latin American migrants in Toronto found that despite relatively high education, participants were food insecure. This finding could be attributed to participants having low-wage positions [58]. Factors that impact migrants’ food access and availability (e.g., shopping practices, food choices) may extend beyond financial and economic status [50,59,60]. Nevertheless, some wealthier families are still food insecure despite being able to read labels. This may be because they are unable to find culturally suitable foods that they prioritise in their diet. Moreover, they may find it difficult to substitute other foods that may be locally available and culturally suitable despite their unfamiliarity. 

### Strengths, Limitations, and Future Directions

This is the first study in Australia to explore the relationship between food labelling and food insecurity among ethnic minority migrants. Some of the strengths of this study include a good sample size and a good response rate. It also has comprehensively explored factors involved in food insecurity and food label knowledge that has not been extensively studied in Australia. Nonetheless, there are several limitations to this study. Generalisability is limited due to the nature of the population studied and the type of sampling adopted. Despite the risk of lower levels of representativeness compared to random sampling, it was necessary to use convenience sampling because of the small number of the difficult-to-reach population of Libyan migrants in Australia. Data may therefore be limited due to selection bias and the possibility that the studied population may not be typical of Libyan families in general. Moreover, the majority of Libyan migrants arrived in Australia as advantaged populations, often highly educated students (some with families) who had not previously experienced food insecurity (unlike typical refugee populations) but found themselves suddenly dispossessed due to a revolution. Thus, not all cultural characteristics of the sample are shared with other Australian migrant and refugee populations. This might limit its generalisability to these groups both here and overseas.

There is abundant space for further investigation on food insecurity and food labelling among migrant and minority populations in Australia. While food security dimensions are broader than financial factors, a relationship with food labelling was not supported by this study. This may be due to the unique population studied, which was generally highly educated and literate. It is therefore recommended that future studies utilise random sampling for increased generalisability and include other populations. The inclusion of interviews would enable further exploration of factors that apply to food insecurity and this population. The use of mixed-method studies would explore all its dimensions and complexities.

## 5. Conclusions

This is the first study to explore food insecurity amongst Libyan migrants. This study contributes to a theoretical understanding about food insecurity and food labelling among a group of migrant families in Australia. In this study, food insecurity was a widespread problem experienced by most families (despite their high education). The number of people in a household, the location of food stores, and the price of food were significantly related to food insecurity. Although labelling comprehension was not an apparent significant factor, more than one-third of the participants had a limited understanding of food labels. This study also revealed that gender, private health insurance, household annual income, education level, and food store type and location were significantly related to the understanding of food labelling. However, it does not mean that food labelling is unimportant, rather other factors have been shown to have greater significance. Nevertheless, simplified labelling could benefit recent migrant populations and others in Australia. This is especially for those attempting to source culturally appropriate foods and acceptable substitutes in their new homeland. Such issues may yet be more fully explored using a culturally sensitive in-depth methods approach. 

To facilitate greater food security among Libyan and other migrant groups, policy makers should consider adopting simplified food labelling. Inclusion of easily recognised symbols for a vegetarian, vegan, and *halal* status of foods could also be beneficial. This would aid food choices that reflect the family requirements of all residents, both minority and majority populations. Education programs that target specific minorities in their most familiar language could increase label comprehension and knowledge of appropriate substitutes for culturally familiar foods that might be expensive or unavailable in their area. Such an approach has the potential to enhance migrant food security. It could be situated within a broad food literacy approach that allows this and other issues related to food security and nutrition among migrants to be addressed.

## Figures and Tables

**Table 1:** Socio-demographic and socio-economic factors of the sample by the level of limited understanding of food labels and the 18-item food insecurity measures.

Factors	Total N (%)			Limited Understanding of Food Label *p*-Value	Food Insecurity *p*-Value
		Limited Understanding of Food Label	Food Insecurity		
		N (%) or Mean (SD)	N (%) or Mean (SD)		
**Socio-Demographic Attributes**					
Age, mean (SD)	38.2 (6.9)	36.9 (7.2)	38.7 (6.6)	0.020	0.041
Gender					
Man Woman	107 (39.5) 164 (60.5)	39 (40.2) 59.8 (59.8)	82 (41.8) 114 (58.2)	0.856	0.200
Length of stay, mean (SD)	8.8 (2.7)	8.24 (2.5)	8.45 (3.0)	0.020	0.247
English language proficiency Low level Intermediate level High level	56 (20.7) 77 (28.4) 138 (50.9)	28 (28.9) 24 (24.7) 45 (46.4)	38 (19.4) 59 (30.1) 99 (50.5)	0.040	0.524
Postcode classification Urban Rural	246 (90.8) 25 (9.2)	86 (88.7) 11 (11.3)	183 (93.4) 13 (6.6)	0.369	0.017
Education Vocational, high school or less Undergraduate university Postgraduate university	45 (16.6) 71 (26.2) 155 (57.2)	28 (28.9) 29 (29.9) 40 (41.2)	36 (18.4) 49 (25) 111 (56.6)	0.000	0.416
Number of family members, mean (SD)	5 (1.6)	4.86 (1.9)	5.2 (1.7)	0.207	0.004
**Socio-Economic Attributes**					
Employment status Yes No	101 (37.4) 169 (62.6)	28 (28.9) 69 (71.1)	73 (37.4) 122 (62.6)	0.030	0.988
Annual income <AUD 40,000 ≥AUD 40,000	139 (64.4) 77 (35.6)	65 (81.3) 15 (18.8)	112 (67.9) 53 (32.1)	0.000	0.052
Occupation status Managerial and professionals * Unskilled, pensioner or unemployed	55 (21) 207 (79)	12 (12.6) 83 (87.4)	40 (21.1) 150 (78.9)	0.012	0.969
Private health insurance Yes No	54 (19.9) 217 (80.1)	26 (26.8) 71 (73.2)	39 (19.9) 157 (80.1)	0.034	0.985
IRSAD ** 1–2 3–4 5–6 7–8 9–10	31 (11.6) 56 (21.0) 40 (15.0) 102 (38.2) 38 (14.2)	11 (11.5) 17 (17.7) 11 (11.5) 39 (40.6) 18 (18.8)	19 (9.7) 40 (20.5) 33 (16.9) 75 (38.5) 28 (14.4)	0.356	0.395
Food store type Supermarket Local food store Supermarket and local store	76 (28.0) 74 (27.3) 121 (44.6)	28 (28.9) 41 (42.3) 28 (28.9)	54 (27.6) 55 (28.1) 87 (44.4)	0.000	0.896
**Food Security Barriers**					
Location of food stores Always/Occasionally Seldom	152 (56.1) 119 (43.9)	64 (66) 33 (34)	130 (66.3) 66 (33.7)	0.014	0.000
Price of food Always/occasionally Seldom	164 (60.5) 107 (39.5)	64 (66.0) 33 (34.0)	137 (69.9) 59 (30.1)	0.170	0.000
Availability of healthy food Always/occasionally Seldom	87 (32.1) 184 (67.9)	32 (33.0) 65 (67.0)	74 (37.8) 122 (62.2)	0.815	0.001
Availability of culturally appropriate foods Always/occasionally Seldom	191 (70.7) 79 (29.3)	73 (76.0) 23 (24.0)	151 (77.4) 44 (22.6)	0.155	0.000
Quality of food Always/occasionally Seldom	105 (38.7) 166 (61.3)	42 (43.3) 55 (56.7)	84 (42.6) 112 (57.1)	0.251	0.025

* Open ended question; ** Index of Relative Socio-economic Advantage and Disadvantage, Postal Area Code (POA) (Ranking within Australia, Socio-Economic Indexes for Areas (SEIFA)). Note: Three postcodes were not found on the 2016 ABS SEIFA (2186, 2610, 3336). Our data were collected at the end of 2019 and the beginning of 2020, therefore, these may be new suburbs.3.2. Univariable Analysis of Food Labelling.

**Table 2:** Univariable and multivariable logistic regression for the understanding of food labels—odds ratio (OR) and 95% confidence interval (CI).

Variable	Univariable Limited Understanding of Food Label N (%)			Multivariable Limited Understanding of Food Label N (%)		
	OR	95% CI	*p*-Value	OR	95% CI	*p*-Value
**Socio-Demographics Attributes**						
Age	1.06	(1.02, 1.10)	0.021	Insignificant in final model		
Gender						
Man Woman	Reference category 0.95	(0.57, 1.58)	0.856	0.50	(0.25, 1.01)	0.049
Length of stay	0.89	(0.81, 1.00)	0.022	Insignificant in final model		
English language proficiency Low level Intermediate level High level	Reference category 0.45 0.48	(0.22, 0.92) (0.28, 0.91)	0.029 0.025	Insignificant in final model		
Post code classification Urban Rural	Reference category 1.46	(0.64, 3.36)	0.371	Insignificant in final model		
Education Vocational, high school or less Undergraduate university Postgraduate university	Reference category 0.42 0.21	(0.19, 0.90) (0.10, 0.43)	0.026 0.000	0.57 0.275	(0.20, 1.63) (0.11, 0.70)	0.013
Number of family members	0.90	(0.77, 1.05)	0.178	Insignificant in final model		
**Socio-Economic Attributes**						
Employment status Working Not working	Reference category 1.80	(1.06, 3.06)	0.031	Insignificant in final model		
Annual income <AUD 40,000 ≥AUD 40,000	Reference category 0.27	(0.14, 0.53)	0.000	0.34	(0.17, 0.72)	0.004
Occupation status * Managerial, professionals or skilled Unskilled, pensioner or unemployed	Reference category 2.41	(1.19, 4.81)	0.014	Insignificant in final model		
Private health insurance Yes No	Reference category 0.52	(0.30, 1.00)	0.036	0.41	(0.19, 1.01)	0.028
IRSAD ** 1–2 3–4 5–6 7–8 9–10	Reference category 0.79 0.69 1.12 1.63	(0.31, 2.0) (0.25, 1.90) (0.49, 2.60) (0.62, 4.33)	0.624 0.472 0.782 0.321	Insignificant in final model		
Food store type Supermarket Local food store Supermarket and local store	Reference category 1.94 4.13	(1.03, 3.60) (2.21, 7.70)	0.039 0.000	1.87 0.51	(0.81, 4.37) (0.23, 1.13)	0.006
**Food Security Barriers**						
Location of food stores Always/occasionally Seldom	Reference category 0.53	(0.31, 0.88)	0.015	2.05	(1.03, 4.09)	0.040
Availability of healthy food Always/occasionally Seldom	Reference category 1.06	(0.63, 1.81)	0.815	Insignificant in final model		
Availability of culturally appropriate foods Always/occasionally Seldom	Reference category 1.50	(0.85, 2.65)	0.156	Insignificant in final model		
Price of food Always/occasionally Seldom	Reference category 1.43	(0.86, 2.40)	0.170	Insignificant in final model		
Quality of food and food storage Always/occasionally Seldom	Reference category 1.34	(0.81, 2.23)	0.251	Insignificant in final model		

* Open ended question. ** Index of Relative Socio-economic Advantage and Disadvantage, Postal Area Code (POA) (Ranking within Australia, Socio-Economic Indexes for Areas (SEIFA)).

**Table 3:** Univariable and multivariable logistic regression for the 18-item food insecurity measure—odds ratio (OR) and 95% confidence interval (CI).

Parameter	Univariable 18-Item N (%)			Multivariable 18-Item N (%)		
	OR	95% CI	*p*-Value	OR	95% CI	*p*-Value
**Socio-Demographics Attributes**						
Age	1.04	(1.0, 1.08)	0.043	Insignificant in final model		
Gender				
Man Woman	Reference category 0.69	(0.40, 1.21)	0.201	Insignificant in final model		
Length of stay	1.06	(0.96, 1.17)	0.247	Insignificant in final model		
English language proficiency Low level Intermediate level High level	Reference category 1.55 1.20	(0.72, 3.35) (0.61, 2.35)	0.263 0.591	Insignificant in final model		
Post code classification Urban Rural	Reference category 0.37	(0.16, 0.86)	0.373	Insignificant in final model		
Education Vocational, high school or less Undergraduate university Postgraduate university	Reference category 0.56 0.63	(0.23, 1.35) (0.28, 1.42)	0.196 0.264	Insignificant in final model		
Number of family members	1.27	(1.07, 1.49)	0.005	1.35	(1.11, 1.63)	0.002
**Socio-Economic Attributes**						
Employment status Employed Unemployed	Reference category 1.00	(0.57, 1.73)	0.988	Insignificant in final model		
Annual income <AUD 40,000 ≥AUD 40,000	Reference category 0.53	(0.28, 1.01)	0.053	Insignificant in final model		
Occupation status * Managerial, professionals or skilled Unskilled, pensioner or unemployed	Reference category 0.99	(0.51, 1.9)	0.969	Insignificant in final model		
Private health insurance Yes No	Reference category 1.01	(0.52, 1.96)	0.985	Insignificant in final model		
IRSAD ** 1–2 3–4 5–6 7–8 9–10	Reference category 1.58 2.98 1.75 1.77	(0.62, 3.99) (1.01, 8.85) (0.75, 4.09) (0.64, 4.91)	0.334 0.050 0.193 0.274	Insignificant in final model		
Food store type Supermarket Local food store Supermarket and local store	Reference category 1.18 1.04	(0.57, 2.42) (0.55, 1.97)	0.653 0.898	Insignificant in final model		
**Food Security Barriers**						
Location of food stores Always/occasionally Seldom	Reference category 4.74	(2.66, 8.46)	0.000	4.53	(2.44, 8.41)	0.000
Availability of healthy food Always/occasionally Seldom	Reference category 2.90	(1.49, 5.62)	0.002	Insignificant in final model		
Availability of culturally appropriate foods Always/occasionally Seldom	Reference category 3.00	(1.71, 5.28)	0.000	Insignificant in final model		
Price of food Always/occasionally Seldom	Reference category 4.128	(2.35, 7.24)	0.000	3.71	(2.44, 8.41)	0.000
Quality of food and food storage Always/occasionally Seldom	Reference category 1.93	(1.08, 3.44)	0.026	Insignificant in final model		
Understanding of food labels Well understanding Limited understanding	Reference category 1.78	(0.99, 3.20)	0.054	Insignificant in final model		

* Open ended question. ** Index of Relative Socio-economic Advantage and Disadvantage, 2016 Postal Area Code (POA) (Ranking within Australia, Socio-Economic Indexes for Areas (SEIFA).

## Data Availability

Not applicable.
